# Adipose-derived stem cells alleviate radiation-induced dermatitis by suppressing apoptosis and downregulating cathepsin F expression

**DOI:** 10.1186/s13287-021-02516-1

**Published:** 2021-08-09

**Authors:** Chaoling Yao, Yue Zhou, Hui Wang, Feiyan Deng, Yongyi Chen, Xiaomei Zhu, Yu Kong, Lijun Pan, Lei Xue, Xiao Zhou, Chunmeng Shi, Xiaowu Sheng

**Affiliations:** 1 Department of Head and Neck Surgery, Central Laboratory, Hunan Cancer Hospital and The Affiliated Cancer Hospital of Xiangya School of Medicine, Tongzipo Road 283, Changsha, 410013 Hunan Province China; 2 Department of Radiation Oncology, Hunan Cancer Hospital and The Affiliated Cancer Hospital of Xiangya School of Medicine, Central South University, Tongzipo Road 283, Changsha, 410013 Hunan Province China; 3 Department of Radiotherapy, National Cancer Center/National Clinical Research Center for Cancer/Cancer Hospital & Shenzhen Hospital, Chinese Academy of Medical Sciences and Peking Union Medical College, Longgang District Baohe Avenue Road 113, Shenzhen, 518116 Guangdong Province China; 4 Nursing Department, Hunan Cancer Hospital and The Affiliated Cancer Hospital of Xiangya School of Medicine, Tongzipo Road 283, Changsha, 410013 Hunan Province China; 5 Institute of Neuroscience, Chinese Academy of Sciences, 320, Yue Yang Road, Shanghai, 200031 China; 6 Pathology Department, Hunan Cancer Hospital and The Affiliated Cancer Hospital of Xiangya School of Medicine, Tongzipo Road 283, Changsha, 410013 Hunan Province China; 7 Institute of Rocket Force Medicine, State Key Laboratory of Trauma, Burns and Combined Injury, Third Military Medical University, No. 30, Main Street, Gaotanyan, Shapingba District, Chongqing, 400038 China

**Keywords:** Adipose-derived stem cells, Apoptosis, Cathepsin F, Radiation-induced dermatitis

## Abstract

**Background:**

Radiation-induced dermatitis is a serious side effect of radiotherapy, and very few effective treatments are currently available for this condition. We previously demonstrated that apoptosis is an important feature of radiation-induced dermatitis and adipose-derived stem cells (ADSCs) are one of the most promising types of stem cells that have a protective effect on acute radiation-induced dermatitis. Cathepsin F (CTSF) is a recently discovered protein that plays an important role in apoptosis. In this study, we investigated whether ADSCs affect chronic radiation-induced dermatitis, and the underlying mechanisms involved.

**Methods:**

ADSCs were isolated from male Sprague-Dawley (SD) rats and characterized. For in vivo studies, rats were randomly divided into control and ADSC-treated groups, and cultured ADSCs were transplanted into radiation-induced dermatitis model rats. The effects of ADSC transplantation were determined by skin damage scoring, histopathological analysis, electron microscopy, immunohistochemical staining, and western blotting analysis of apoptosis-related proteins. To evaluate the effects of ADSCs in vitro, radiation-induced apoptotic cells were treated with ADSC culture supernatant, and apoptosis-related protein expression was investigated by TUNEL staining, flow cytometry, and western blotting.

**Results:**

In the in vivo studies, skin damage, inflammation, fibrosis, and apoptosis were reduced and hair follicle and sebaceous gland regeneration were enhanced in the ADSC group compared with the control group. Further, *CTSF* and downstream pro-apoptotic proteins (Bid, BAX, and caspase 9) were downregulated, while anti-apoptotic proteins (Bcl-2 and Bcl-XL) were upregulated. In vitro, ADSCs markedly attenuated radiation-induced apoptosis, downregulated *CTSF* and downstream pro-apoptotic proteins, and upregulated anti-apoptotic proteins.

**Conclusion:**

ADSCs protect against radiation-induced dermatitis by exerting an anti-apoptotic effect through inhibition of *CTSF* expression. ADSCs may be a good therapeutic candidate to prevent the development of radiation-induced dermatitis.

## Background

Radiation-induced dermatitis is a serious side effect of radiotherapy, up to 95% of patients who undergo radiotherapy develop dermatitis. Acute radiation-induced dermatitis occurs within a few days after radiotherapy and is characterized by erythema and desquamation [1, 2]. Chronic radiation-induced dermatitis usually occurs more than 90 days after radiotherapy and is characterized by fibrosis and thickening of the dermis [3, 4]. Importantly, radiation-induced dermatitis may limit the radiation dose delivered and even necessitate treatment termination. Currently, this condition is treated using drugs, wound dressing, and even surgical treatment [1, 5]. However, these interventions have various limitations, such as poor efficacy or high risk; therefore, new effective treatments are urgently needed.

In our previous study, we established a rat model of radiation-induced dermatitis and found that apoptosis is an important feature of this condition [6]. Apoptosis is a major route of radiation-induced cell death. Radiation can directly break chemical bonds in the DNA backbone, creating single- and double-strand breaks, thus causing DNA damage in irradiated cells. Double-strand breaks in normal cells activate ataxia telangiectasia-mutated and DNA-dependent protein kinase [7], which phosphorylate histone H2AX and P53-binding protein 53BP1 and guide cells toward repair or ultimately induce cell cycle arrest and apoptosis [8]. Moreover, ionizing radiation interacts with water molecules to form reactive oxygen species, such as superoxide, hydrogen peroxide, and hydroxyl radicals [9]. Reactive oxygen species activate several signaling pathways, including p53-mediated apoptosis, mitogen-activated protein kinase signaling, nuclear factor E2-related factor 2 signaling, hypoxia-inducible factor-1α signaling, and nuclear factor κΒ signaling [10], and induce cell injury by damaging proteins and nucleic acids. Injured cells release various inflammatory chemokines and cytokines and attract inflammatory cells to the site of injury, thus facilitating the release of more proinflammatory cytokines, such as tumor necrosis factor-α, interleukin (IL)-1, interferon-γ, and IL-6 [11]. Increased levels of these proinflammatory cytokines activate pro-apoptotic signals and induce apoptosis [12].

Cathepsin F (CTSF) is a recently described papain-like cysteine protease that plays an important role in the apoptotic process [13]. *CTSF* expression is downregulated in gastric cancer cells and tissues, and *CTSF* knockdown significantly suppressed gastric cancer cell apoptosis [14]. KEGG pathway database analysis revealed that *CTSF* reduces Bcl-2 in the apoptotic pathway [14]. Janic et al. showed that *CTSF* knockdown induced leukemia development [15]. However, whether *CTSF* is involved in the pathogenesis of radiation-induced dermatitis is unclear.

Adipose-derived stem cells (ADSCs) are a type of mesenchymal stem cells that are derived from adipose tissue. They can readily adhere to plastic culture flasks, are easily expanded in vitro, and are considered to have broad clinical application potential. ADSCs were first isolated by Zuk et al. [16]. ADSCs can differentiate into multiple cell lineages and secrete various paracrine factors. For example, they secrete hepatocyte growth factor, IL-12, and superoxide dismutase (an enzyme with anti-oxidant effects); vascular-derived and platelet-derived growth factors to exert pro-angiogenic effects; stromal-derived factor-1 to recruit non-injured stem cells to the injury site; and insulin-like growth factor-1 to promote hair regeneration [17]. Furthermore, exosomes from ADSCs can reduce neuronal cell apoptosis [18]. ADSCs have been shown to exhibit anti-inflammatory effects in a bleomycin-induced interstitial pneumonia mouse model [19] and to attenuate pulmonary fibrosis of silicosis via anti-inflammatory and anti-apoptotic effects [20]. We have previously demonstrated that ADSCs have a protective effect on acute radiation-induced dermatitis [21]. However, whether they have a similar effect on chronic radiation-induced dermatitis and the underlying mechanisms remain unclear.

Therefore, in this study, we evaluated the effects of ADSCs on acute and chronic radiation-induced dermatitis, and we preliminarily analyzed their underlying mechanism.

## Methods

### Experimental animals and wound assessment after irradiation

Sprague-Dawley (SD) rats weighing 220–250 g were obtained from Hunan SJA Laboratory Animal Co., Ltd. (Hunan, China). All researchers who performed the animal operations received training in animal experiments from Central South University. Ethics approval was obtained from the Animal Ethics Committee of Hunan Cancer Hospital and the Affiliated Cancer Hospital of Xiangya School of Medicine, Central South University. To establish the radiation-induced dermatitis model, SD rats were irradiated as described previously [6]. The rats were anesthetized by pentobarbital sodium (5%, 50 mg/kg) injection. Forty-eight female SD rats were randomly divided into control and ADSC groups (*n* = 24 per group). Rats in the control group received 90 Gy of irradiation and injected with PBS, whereas those in the ADSC group were irradiated with 90 Gy and then injected with 10^7^ ADSCs within 24 h. The degree of skin tissue toxicity after irradiation was scored independently by two individuals who were blinded to the groups, according to previous reports [22, 23]. Wound scores and pictures were recorded weekly until 12 weeks after the operation.

### Isolation, differentiation, and characterization of rat ADSCs

Inguinal adipose tissue was harvested from male rats after they were euthanized. Subcutaneous visible blood vessels were removed, and the tissue was rinsed with phosphate-buffered saline (PBS) (Hyclone, Logan, UT, USA), cut into pieces, and subjected to enzymatic digestion with 1 μg/ml type I collagenase (Invitrogen, Carlsbad, CA, USA) in a constant-temperature water bath at 37 °C for 60 min. After neutralization of the collagenase using cell culture medium, the dispersed tissue was centrifuged at 1000 × *g* at 25°C for 10 min. The floating fat was aspirated, and the cell suspension was filtered through a 70-μm screen mesh to remove tissue fragments. The filtrate was then centrifuged at 1000×*g* for 10 min, the supernatant was discarded, and the cells were collected and cultured in Dulbecco’s modified Eagle’s medium (DMEM, Gibco, Grand Island, NY, USA) supplemented with 10% fetal bovine serum (Gibco, Carlsbad, CA, USA) and 1% penicillin/streptomycin (Gibco, Grand Island, NY, USA) at 37°C in a 5% CO_2_ atmosphere. For cell differentiation and identification, ADSCs from passage 3 were used. In vitro differentiation of ADSCs was induced using adipogenic, chondrogenic, and osteogenic differentiation media (Cyagen, Chicago, IL, USA) in six-well plates. ADSC differentiation into adipocytes was induced for 20 days, and differentiation into chondrocytes and osteoblasts for 21 days. Differentiated cells were fixed and stained with oil red O, alcian blue, or alizarin red and observed under a microscope (Carl Zeiss, Oberkochen, Germany). The expression of ADSC immunomarkers, including CD10, CD34, CD45, CD73, CD90, and CD105 (Abcam, Cambridge, UK), was evaluated by flow cytometry.

### Cell culture, irradiation, and treatments

Human immortalized keratinocytes (HaCaT; Wuhan Yipu Biological Technology Co., Ltd., Wuhan, China) and normal human oral squamous epithelial cells (NOK; Shanghai Binsui Biological Technology Co., Ltd., Shanghai, China) were cultured in DMEM supplemented with 10% fetal bovine serum and 1% streptomycin/penicillin. HaCaT and NOK cells were irradiated with a linear accelerator (Varian Rapid ARC, Palo Alto, CA, USA) at doses of 20 and 5 Gy, respectively. The dose rate was 600 Mu/min. ADSC culture supernatant was collected and centrifuged at 1000×*g* for 5 min to remove cell debris. Irradiated cells were immediately treated with ADSC culture supernatant. Irradiated cells treated with normal culture medium served as a control. The medium was changed every 24 h. Cells were collected 72 h after irradiation for apoptosis and for western blotting assays.

### Histological staining

Skin tissues were fixed in 4% paraformaldehyde, paraffin-embedded, and sectioned at 4-μm thickness. The sections were routinely stained with hematoxylin and eosin (Servicebio, Wuhan, China) and Masson’s trichrome (Servicebio, Wuhan, China). The cells were analyzed and imaged under an optical microscope (Carl Zeiss, Oberkochen, German).

### Hydroxyproline content assay

To quantify collagen in skin tissues, the hydroxyproline content was measured using a hydroxyproline assay kit (Jiancheng Bioengineering Institute, Nanjing, China) following the manufacturer’s protocol, as previously reported [6]. The hydroxyproline content was normalized to wet tissue weight.

### Immunohistochemical staining

Immunohistochemical staining was performed as described previously [6]. BAX polyclonal antibody (1:200; 50599-2-Ig), Bcl2 polyclonal antibody (1:200; 26593-1-AP), and *CTSF* polyclonal antibody (1:200; 11055-1-AP) were all purchased from ProteinTech (Chicago, IL, USA). To quantify protein levels, the average optical density (OD) value of positive staining was determined using the Image-Pro Plus software.

### Electron microscopy

Skin tissues were fixed with 2.5% glutaraldehyde in phosphate buffer and sliced into 50-μm sections. The ultrastructure of skin cells and organelles was evaluated using an electron microscope (FEI, Hillsboro, OR, USA).

### Terminal deoxynucleotidyl transferase dUTP neck-end labeling (TUNEL) staining

TUNEL staining was performed using a TUNEL Apoptosis Assay Kit (G1501; Servicebio, Wuhan, China), as described previously [6]. TUNEL^+^ cells were counted manually under an inverted fluorescence microscope (Carl Zeiss, Oberkochen, German).

### Flow-cytometric detection of apoptosis

Apoptosis of NOK and HaCaT cells after irradiation and treatment with ADSC culture supernatant was detected using an Annexin V-APC/PI apoptosis detection kit (KG, Nanjing). Briefly, cells were harvested, washed twice with PBS, and resuspended in binding buffer. Annexin V-APC and PI were added, and the cells were incubated at room temperature in the dark for 10 min and then analyzed within an hour using a flow cytometer (Beckman, Hialeah, FL, USA).

### Small interfering (si)RNA transfection

SiRNAs targeting *CTSF* and negative control siRNA (Table 1) were synthesized at GenePharma (Shanghai, China). HaCaT cells were transfected using GP-transfect-Mate Transfection Reagent (Gene Pharma, Shanghai, China) according to the manufacturer’s protocol.

**Table 1:** SiRNAs used in this study

*Homo sapiens CTSF*	Sense (5′–3′)	Antisense (5′–3′)
*CTSF*-001	GGCUCAGCCAUGAUUUCUUTT	AAGAAAUCAUGGCUGAGCCTT
*CTSF*-002	CCAUCAAUGCCUUUGGCAUTT	AUGCCAAAGGCAUUGAUGGTT
Negative control	UUCUCCGAACGUGUCACGUTT	ACGUGACACGUUCGGAGAATT

### Western blotting

Proteins were extracted from skin tissues and cells using RIPA lysis buffer and were quantified using the bicinchoninic acid method. The proteins were subjected to sodium dodecyl sulfate-polyacrylamide gel electrophoresis and transferred to nitrocellulose membranes. The membranes were blocked in 5% nonfat milk at room temperature for 1 h. Next, they were incubated with antibodies against *CTSF* (0.1 μg/mL; R&D, AF2075, Minneapolis, MN, USA), Bid (1:1000; 10988-1-AP, ProteinTech, USA), BAX (1:5000; 50599-2-Ig, ProteinTech, USA), Bcl-2 (1:500; 26593-1-AP, ProteinTech, USA), Bcl-XL (1:1000; 10783-1-AP, ProteinTech, USA), caspase 9 (1:300; 10380-1-AP, ProteinTech, USA), and GAPDH (1:5000; 10494-1-AP, ProteinTech, USA) at 4 °C overnight. After washing, the membranes were incubated with horseradish peroxidase-labeled goat anti-rabbit IgG (1:6000; SA00001-2, ProteinTech, USA) for 2 h. The blots were developed using enhanced chemiluminescence, and band densities were analyzed using the Quantity One software (Bio-Rad, Hercules, CA, USA).

### Statistical analysis

All data are presented as the mean ± standard error of the mean and were analyzed using SPSS (version 13.0; SPSS, Chicago, IL, USA). Differences between groups were analyzed using Student’s *t*-test; *p* < 0.05 was considered statistically significant.

## Results

### Isolation and characterization of ADSCs

**Fig. 1:**
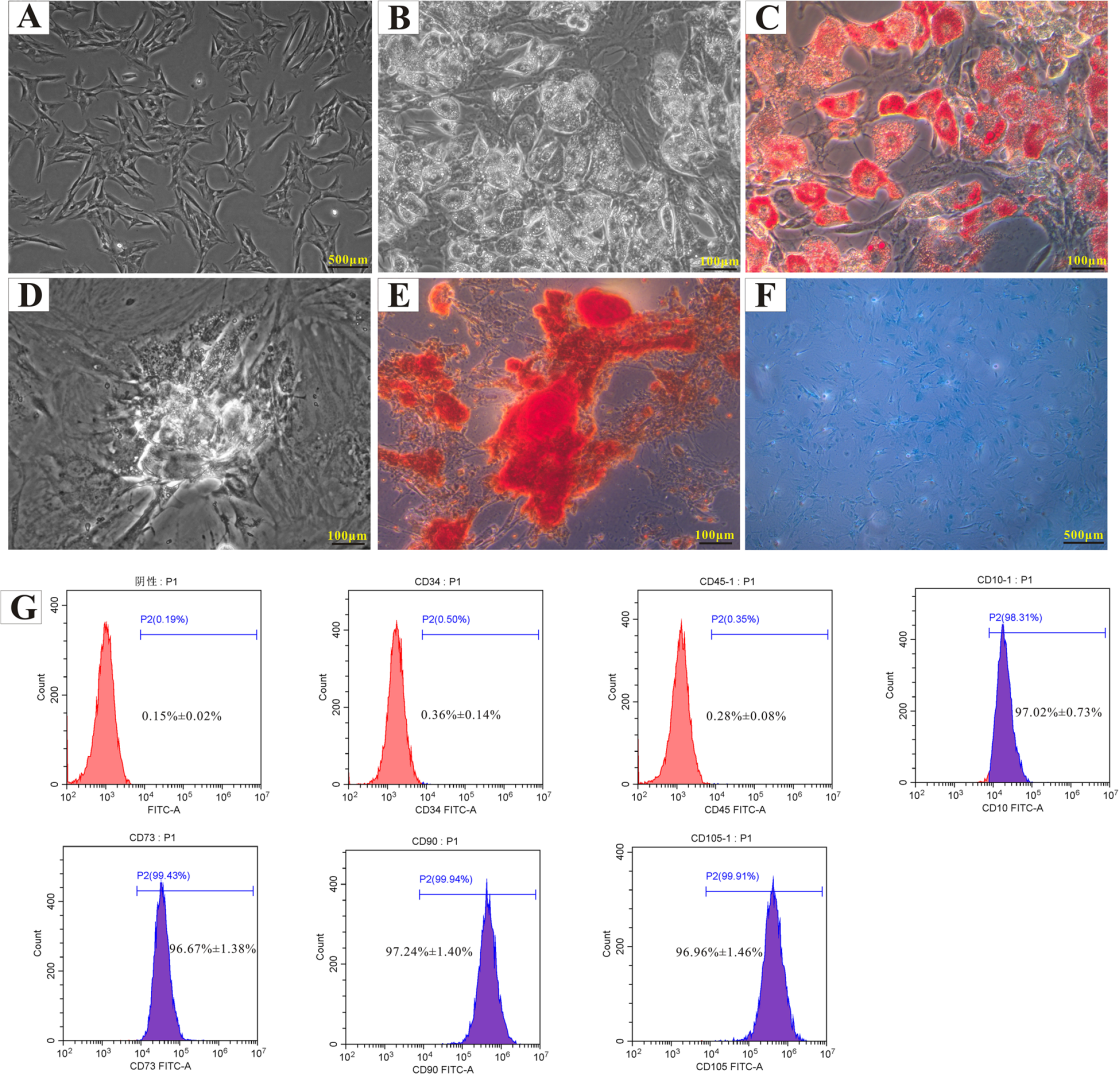
Isolation and characterization of ADSCs. ADSCs isolated from male rat adipose tissue exhibited a typical spindle shape in culture (**A**). The multilineage differentiation potential of the ADSCs was investigated: adipogenesis was demonstrated by oil red O staining (**B, C**), osteogenesis by alizarin red S staining (**D, E**), and chondrogenesis by alcian blue staining (**F**). The expression of ADSC surface markers was evaluated by flow cytometry (**G**)

ADSCs were isolated from groin adipose tissues of male rats. Cultured ADSCs had a spindle shape (Fig. 1A) and adipogenic (Fig. 1B, C), osteogenic (Fig. 1D, E), and chondrogenic (Fig. 1F) differentiation potential. The ADSCs were analyzed for the expression of specific surface markers; they were strongly positive for CD10 (97.02% ± 0.73%), CD73 (96.67% ± 1.38%), CD90 (97.24% ± 1.40%), and CD105 (96.96% ± 1.46%), but negative for CD34 (0.36% ± 0.14%) and CD45 (0.28% ± 0.08%) (Fig. 1G).

### ADSCs alleviate radiation-induced acute skin reaction

**Fig. 2:**
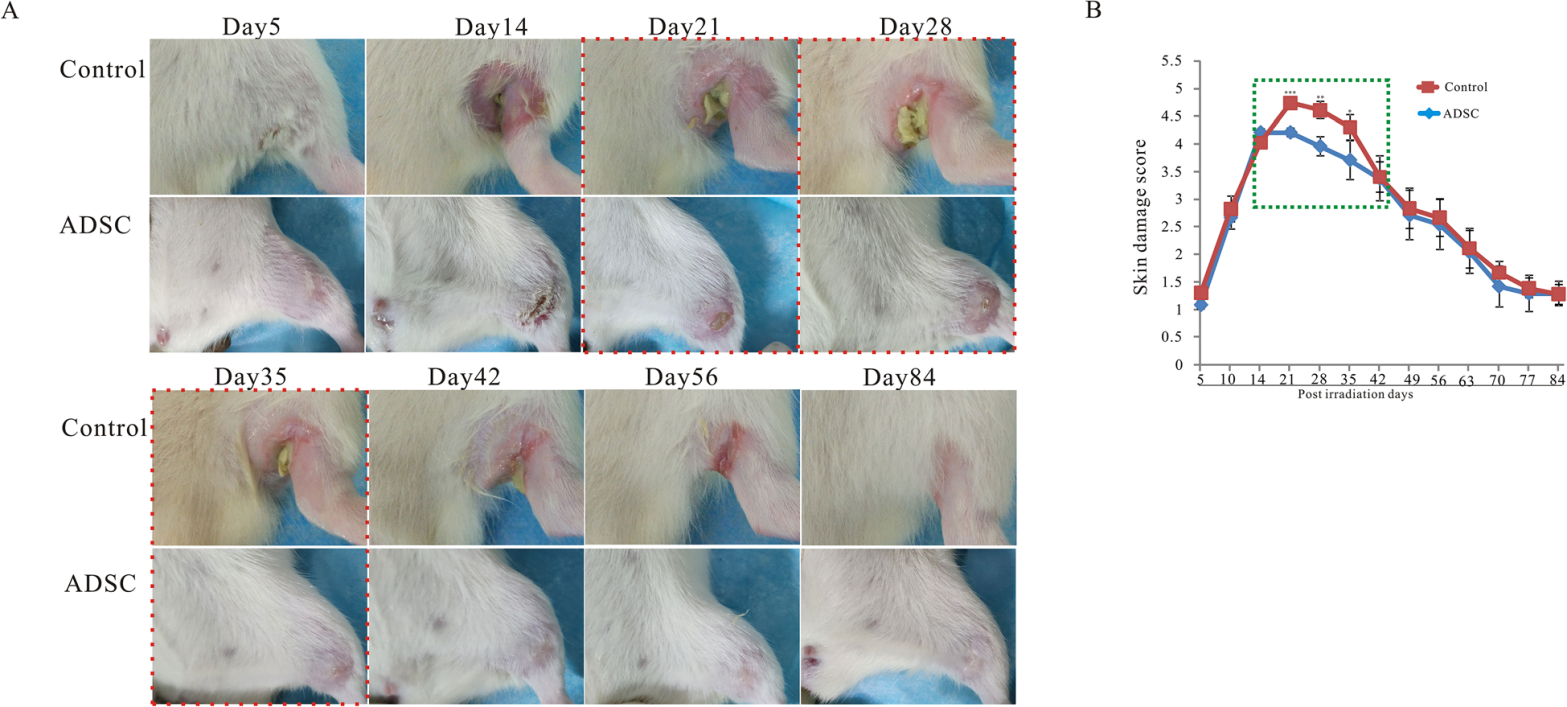
ADSCs alleviate radiation-induced skin damage in radiation-induced dermatitis rat model. Female rats were irradiated with 90 Gy and injected with PBS (control group, *n* = 12) or were irradiated with 90 Gy and injected with 10^7^ ADSCs (ADSC group, *n* = 12). Representative images of the skin from day 5 to day 84 post-irradiation are shown. Skin damage was semiquantitatively scored from 1 (no damage) to 5 (severe damage). **A** Rats in the ADSC group had smaller ulcers and open wounds at 3, 4, and 5 weeks post-irradiation than the control rats (red dotted line) (*n* = 12). **B** Skin damage scores were lower in the ADSC group than in the control group at 3 to 5 weeks post-irradiation, as indicated by the green dotted line (*n* = 12). The results are expressed as the mean ± SEM. *p < 0.05, **p < 0.01, ***p < 0.001 vs. the control group

In the control group, visible skin damage was detected from 5 days post-irradiation. At 2 weeks post-irradiation, the skin showed remarkable erythema, desquamation, and hair loss. At 3 weeks post-irradiation, large ulcers, open wounds, and full-thickness skin loss were observed. At 8 weeks post-irradiation, most of the visible skin damage was repaired by scar tissue. In the ADSC group, visible skin damage was detected at the same time points as in the control group. At 2 weeks post-irradiation, the skin showed erythema and desquamation, but less hair loss than in the control group. At 3, 4, and 5 weeks post-irradiation, the skin exhibited smaller ulcers and open wounds than in the control group (Fig. 2A, indicated by a red dotted line). Most of the visible skin damage was repaired by scar tissue at 4 weeks post-irradiation, which was earlier than in the control group (Fig. 2A). Skin injury scores during the observation period are shown in Fig. 2B; the scores were lower in the ADSC group than in the control group from 3 to 5 weeks post-irradiation (Fig. 2B, green dotted line).

### ADSCs alleviate acute and chronic radiation-induced dermatitis

**Fig. 3:**
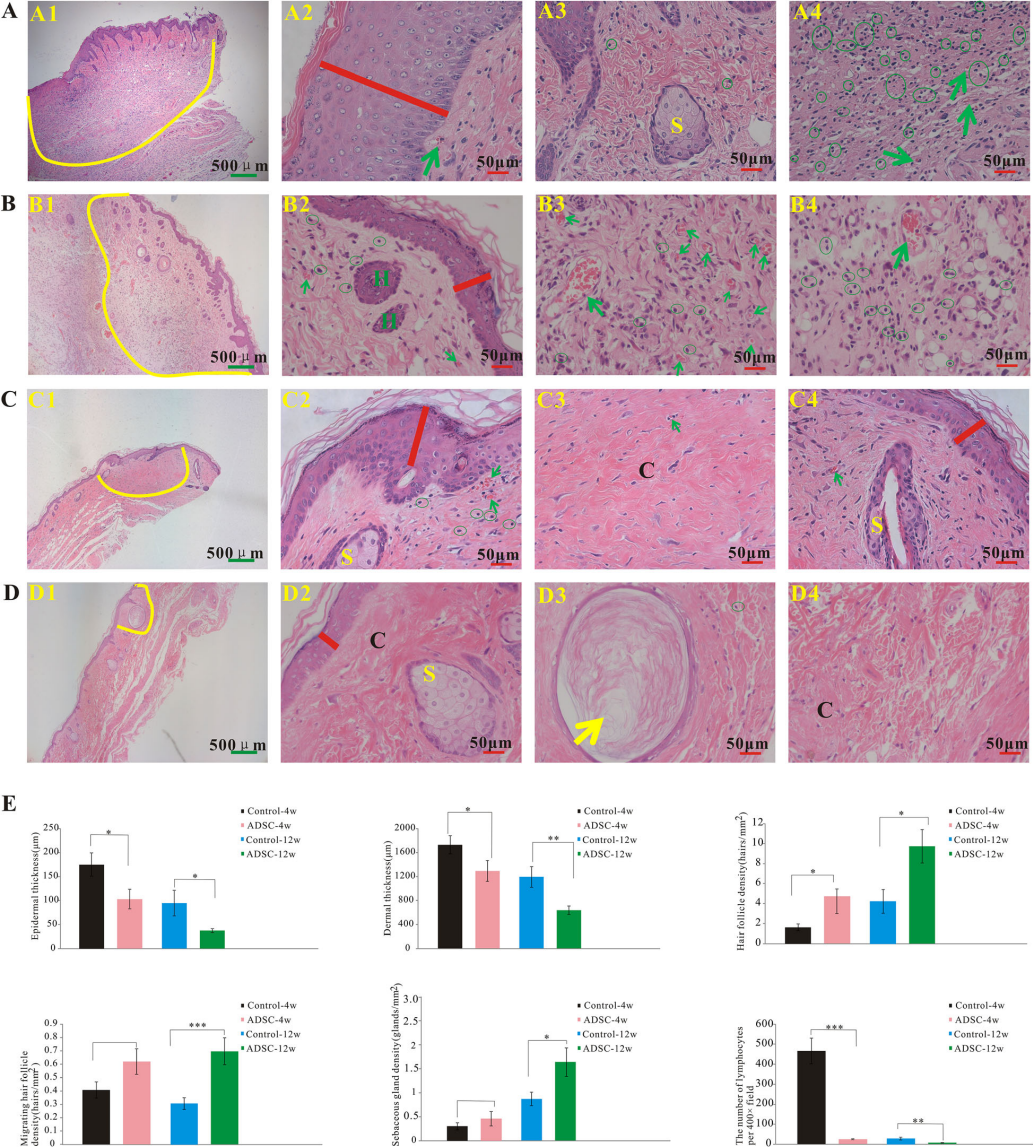
ADSCs alleviate radiation-induced dermatitis in vivo. Representative images of hematoxylin and eosin-stained skins from rats in the control group at 4 weeks (**A**) and 12 weeks post-irradiation (**C**) and the ADSC group at 4 weeks (**B**) and 12 weeks post-irradiation (**D**). Yellow curves indicate skin appendage loss. The red line indicates epidermal thickness. The green arrows indicate blood vessels. Green circles mark infiltrated lymphocytes. Statistical analysis of epidermal thickness, dermal thickness, hair follicle density, migrating hair follicle density, sebaceous gland numbers, and lymphocyte number per 400× field in each group (**E**) (*n* = 8/group). The results are expressed as the mean ± SEM. *p < 0.05, **p < 0.01, ***p < 0.001 vs. the control group. *H* hair follicle, *S* sebaceous gland, *C* collagen

Skin tissues from rats of the control and ADSC groups were collected at 4 and 12 weeks post-irradiation for histological analysis (Fig. 3A–D). In the control group, at 4 weeks post-irradiation, epidermal loss (Fig. 3A (1)), epidermal thickening (Fig. 3A2, red line), lymphocyte infiltration (Fig. 3A (3, 4), green circle), few blood vessels (Fig. 3A (2, 4), green arrow), and skin appendage loss (Fig. 3A (1), yellow curve) were observed. By contrast, at the same time point, the ADSC group showed no epidermal loss, less epidermal thickening (Fig. 3B (2), red line), low lymphocyte infiltration (Fig. 3B (3, 4), green circle), numerous blood vessels (Fig. 3B (3, 4), green arrow), and less skin appendage loss (Fig. 3B (1), yellow curve). At 12 weeks post-irradiation, in the control group, considerable dermal fibrosis with appendage loss (Fig. 3C (1), yellow curve), low lymphocyte infiltration (Fig. 3C (2), green circle), and few blood vessels (Fig. 3C (2, 4), green arrow) were observed. At the same time point, the ADSC group showed less dermal fibrosis than the control group (Fig. 3D (1), yellow circle), and keratinization (Fig. 3D (3)).

Histological analysis of tissue sections confirmed that epidermal thickness was significantly lower in the ADSC group than in the control group at both 4 and 12 weeks post-irradiation (Fig. 3E). Similarly, dermal thickness was significantly lower in the ADSC group than in the control group at both 4 and 12 weeks post-irradiation (Fig. 3E). Hair follicle density was higher in the ADSC group than in the control group at both 4 and 12 weeks post-irradiation compared (Fig. 3E). Migrating hair follicle density was slightly, albeit not significantly, higher in the ADSC group than in the control group at 4 weeks post-irradiation (Fig. 3E) and was significantly lower at 12 weeks post-irradiation in the ADSC group than in the control group (Fig. 3E). Sebaceous gland density was slightly, albeit not significantly, higher in the ADSC group than in the control group at 4 weeks post-irradiation (Fig. 3E) and was significantly lower at 12 weeks post-irradiation in the ADSC group than in the control group (Fig. 3E). The number of lymphocytes per 400× field was significantly lower in the ADSCs rats than in the control rats at 4 and 12 weeks post-irradiation (Fig. 3E).

**Fig. 4:**
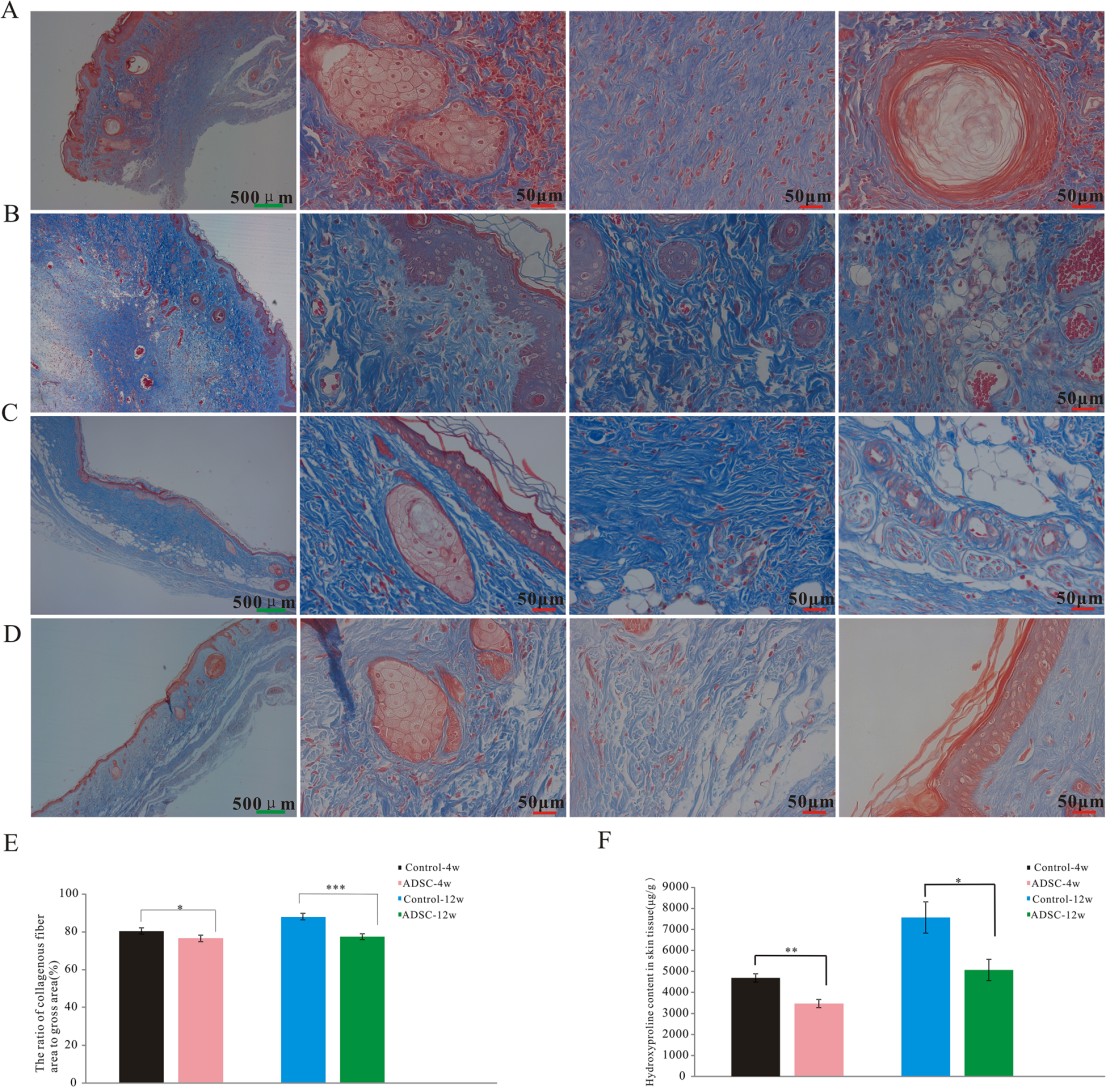
ADSCs suppress skin fibrosis after irradiation in vivo. Representative images of Masson’s trichrome-stained skins from rats in the control group at 4 weeks (**A**) and 12 weeks post-irradiation (**C**) and the ADSC group at 4 weeks (**B**) and 12 weeks post-irradiation (**D**). Collagenous fiber area-to-gross area ratio in each group (**E**) (*n* = 8/group). Fibrosis-related amino acid (hydroxyproline) levels in each group (**F**) (*n* = 6/group). The results are expressed as the mean ± SEM. *p < 0.05, **p < 0.01, ***p < 0.001 vs. the control group

Fibrosis was analyzed using Masson’s trichrome staining (Fig. 4A–D) and by measuring the hydroxyproline content in skin tissues. The collagen fiber percentage was lower in the ADSC group than in the control group at both 4 and 12 weeks post-irradiation (Fig. 4E). The hydroxyproline content of skins of rats of the ADSC group was significantly lower than that in control rats at 4 and 12 weeks post-irradiation (Fig. 4F).

### ADSCs inhibit apoptosis in irradiated skin tissue

**Fig. 5:**
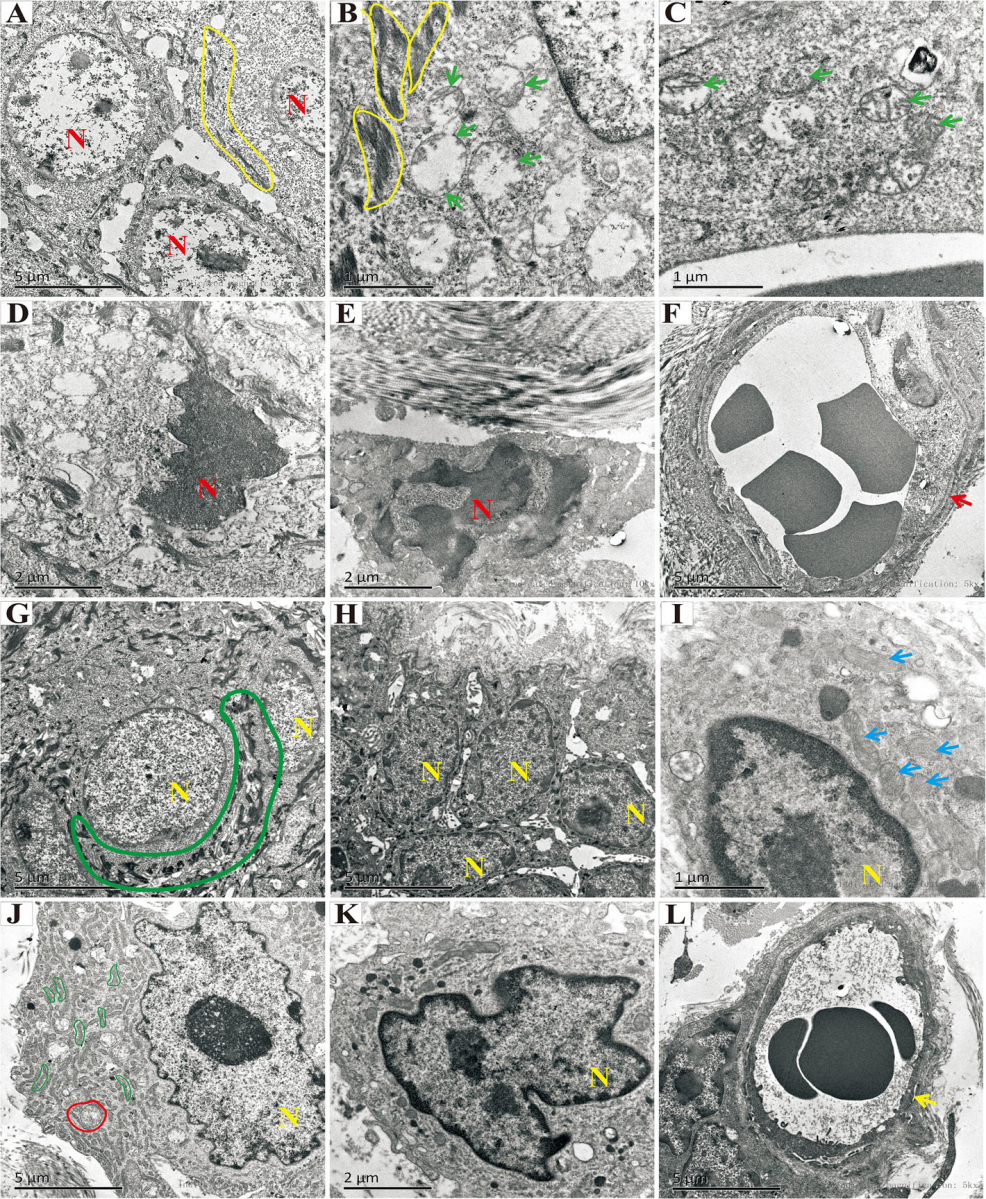
Transmission electron microscopic analysis of the ultrastructure of skin tissues or rats in the control group at 4 weeks post-irradiation (**A–F**) and the ADSC group at 4 weeks post-irradiation (**G**–**L**). Yellow curves indicate irregular desmosomes (**A**, **B**). Green arrows indicate damaged mitochondria, with loss of cristae structures (**B**, **C**). The red “N” indicates nuclear pyknosis and dissolution (**D**–**F**). Large green curves indicate regular desmosomes (**G**), and the yellow “N” indicates regular nuclei in the ADSC group at 4 weeks post-irradiation (**G**–**L**). Blue arrows indicate mitochondria with few cristae (**I**). Small green curves indicate the endoplasmic reticulum (**J**). Yellow arrows indicate regularly shaped vascular endothelium (**L**)

Transmission electron microscopy showed squamous cells with irregular desmosomes (Fig. 5A, B, yellow curve), damaged mitochondria with a loss of cristae (Fig. 5B, C, green arrow), nuclear pyknosis and dissolution (Fig. 5D, E, indicated with “N”), and vascular endothelium swelling (Fig. 5F, red arrow) in skin tissues of rats of the control group at 4 weeks post-irradiation. However, in the ADSC group, at 4 weeks post-irradiation, squamous cells with regular desmosomes (Fig. 5G, large green curve), a large endoplasmic reticulum (Fig. 5J, small green curve), a well-formed autophagic vacuole (Fig. 5J, red circle), mitochondria with a few cristae left (Fig. 5I, blue arrow), regular nuclei (Fig. 5G–L, indicated with “N”), and a regularly shaped vascular endothelium (Fig. 5L, yellow arrow) were observed.

**Fig. 6:**
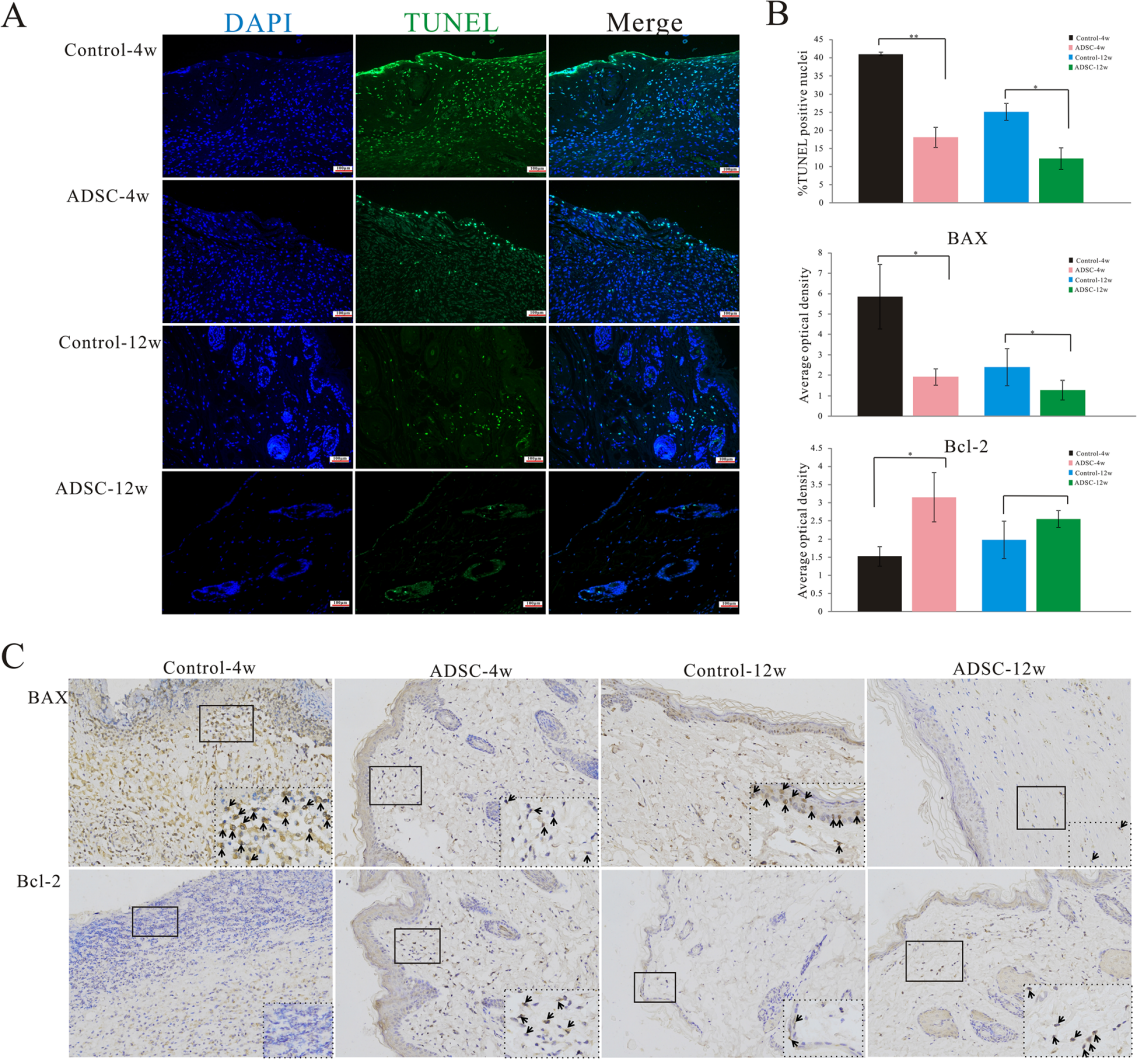
ADSCs inhibit apoptosis in irradiated skin tissue. Representative images of TUNEL-stained cells (**A**). Nuclei of TUNEL-positive cells are stained green (original magnification, 200×). Percentages of apoptotic cells were significantly decreased in the ADSC group at both 4 and 12 weeks post-irradiation compared with those in the control group. Average OD for analysis of expression of apoptosis-related proteins, BAX and BCL2 (**B**) (*n*=6/group). Representative images of skin tissues immunohistochemically stained for BAX and BCL-2 (**C**). Black arrows indicate positive expression of BAX and BCL-2. The results are expressed as the mean ± SEM. *p < 0.05, **p < 0.01 vs. the control group

Representative fluorescence photomicrographs of TUNEL-positive apoptotic cells are shown in Fig. 6A. The percentage of apoptotic cells was significantly lower in the ADSC group than in the control group at both 4 and 12 weeks post-irradiation (Fig. 6B). Representative immunohistochemical staining images are shown in Fig. 6C. The expression of the anti-apoptotic protein Bcl-2 was significantly higher in the ADSC group than in the control group at both 4 and 12 weeks post-irradiation, whereas that of the pro-apoptotic protein BAX was markedly lower in rats of the ADSC group than in control rats at these two time points. Collectively, these findings suggested that ADSCs inhibit apoptosis in irradiated skin tissues in vivo*.*

### ADSCs inhibit apoptosis in irradiated epidermal cells

**Fig. 7:**
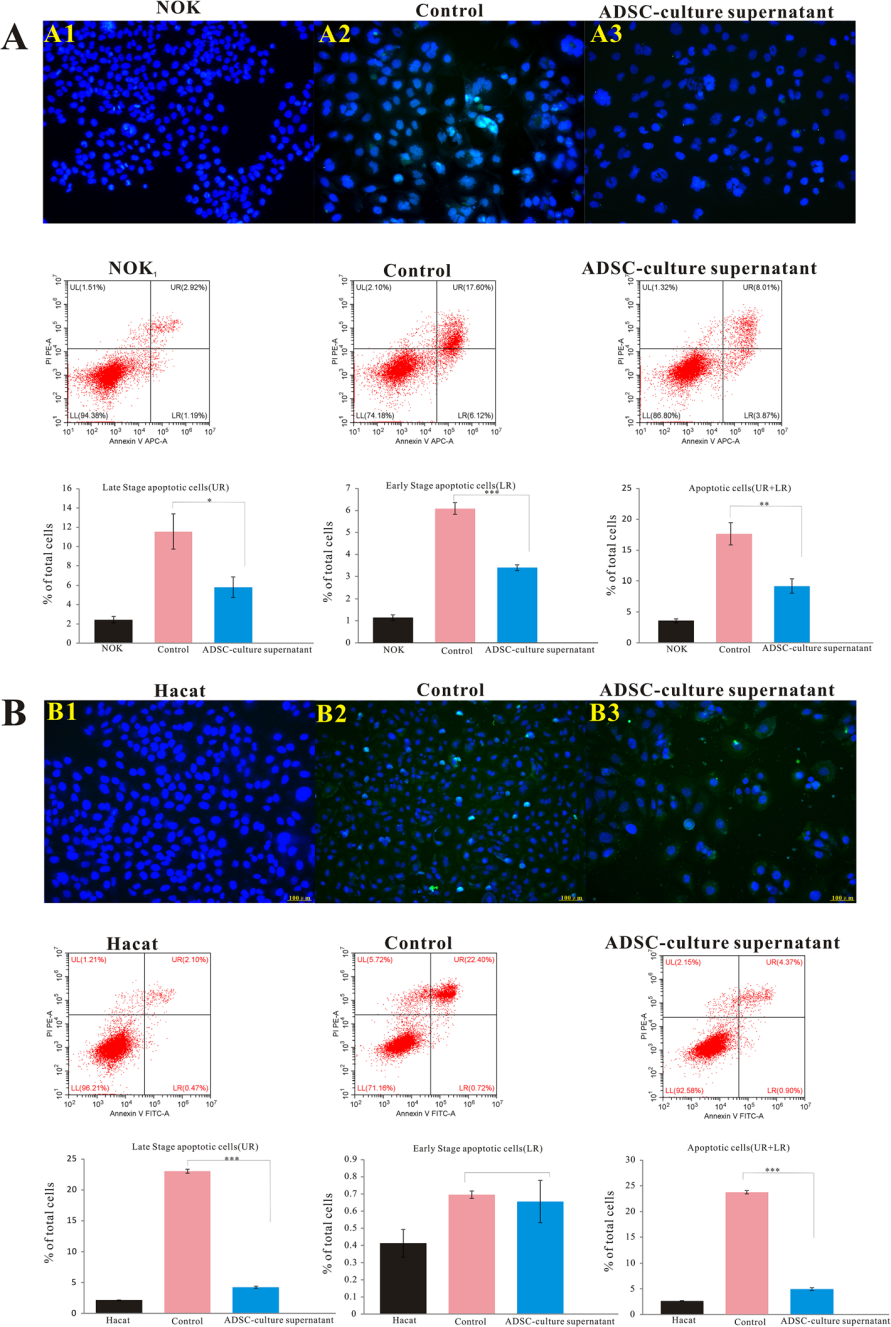
ADSCs inhibit apoptosis in irradiated epidermal cells in vitro. NOK and HaCaT cells were treated with ADSC culture supernatant for 72 h and were then harvested for the detection of apoptosis using flow cytometry. Upper left quadrant—necrotic cells; upper right quadrant—late apoptotic cells; lower left quadrant—viable cells; lower right quadrant—early apoptotic cells. Representative fluorescence photomicrographs of TUNEL-positive apoptotic cells are shown (**A**, **B**). The results are expressed as the mean ± SEM. *p < 0.05, **p < 0.01, ***p < 0.001 vs. the control group

Considering the remarkable apoptosis-inhibitory effect of ADSCs in vivo and that paracrine action is the main function of ADSCs, we further analyzed the effect of ADSCs on apoptosis in vitro. We first established radiation-induced apoptosis in HaCaT and NOK cells. Then, the irradiated cells were treated with ADSC culture supernatant. At 72 h post-irradiation, apoptosis was analyzed by flow cytometry and TUNEL staining (Fig. 7A, B). Flow-cytometric data showed that the proportions of NOK cells in early, late, and total apoptosis were lower in the ADSC culture supernatant-treated group than in the control group (Fig. 7A). Similarly, the proportions of HaCaT cells in late and total apoptosis were lower in the supernatant-treated group than in the control group, whereas the early apoptotic cell proportion did not differ significantly (Fig. 7B). Quantification by flow cytometry showed that ADSC culture supernatant could markedly decrease the total apoptosis ratio. Representative fluorescence photomicrographs of TUNEL-positive apoptotic cells in each group are shown in Fig. 7A1–3 and 7B1–3. The findings suggested that ADSCs inhibit apoptosis in irradiated cells in vitro.

### ADSCs downregulate *CTSF* expression in vivo and in vitro

**Fig. 8:**
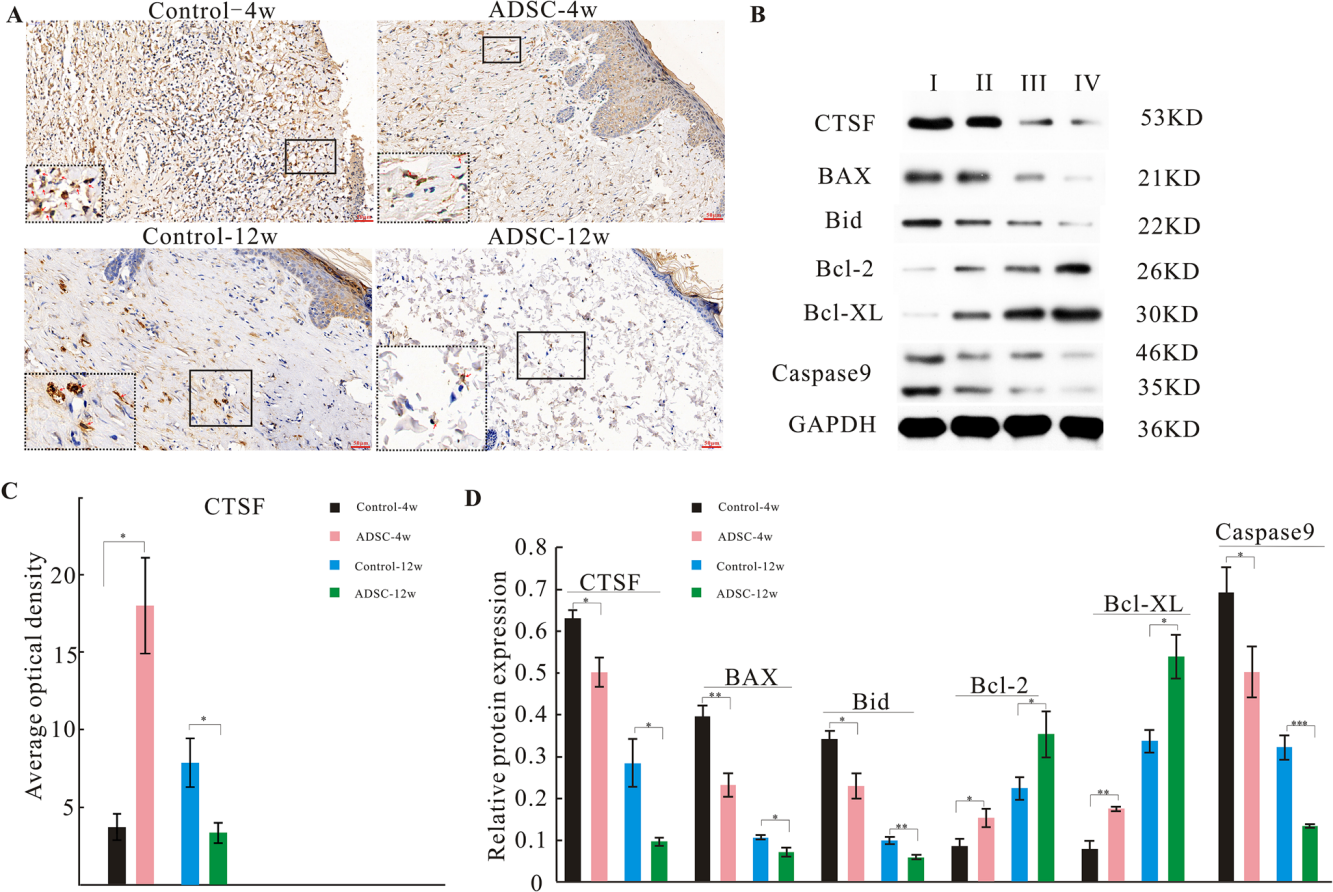
ADSCs downregulate the expression of *CTSF*, BAX, Bid, and caspase 9 and upregulate that of Bcl-2 and Bcl-XL in radiation-induced dermatitis model rats in vivo. Representative images of skin tissues immunohistochemically stained for *CTSF* (**A**). Representative blots of *CTSF*, BAX, Bid, Bcl-2, Bcl-XL, and caspase 9 levels in skin tissues (**B**). GAPDH was used as a loading control. Average OD of *CTSF* (**C**) (*n* = 6/group). Protein expression level analysis (**D**) (*n*=4/group). I, control-4w; II, ADSCs-4w; III, control-12w; IV, ADSCs-12w. The results are expressed as the mean ± SEM. *p < 0.05, **p < 0.01, ***p < 0.001 vs. the control group

We evaluated *CTSF* expression in skin tissues by immunohistochemical staining in vivo (Fig. 8A). At 4 weeks post-irradiation, *CTSF* expression was increased in the control group, whereas this increase was significantly attenuated in the ADSC group (Fig. 8A, red arrows). At 12 weeks post-irradiation, *CTSF* was expressed at low levels in the control group, whereas in the ADSC group, *CTSF* expression was restored to nearly normal levels. Analysis of the average OD value confirmed that *CTSF* expression was significantly lower in the ADSC group at both 4 and 12 weeks post-irradiation than in the control group at the same time points (Fig. 8C).

Next, the expression of *CTSF* and downstream pro-apoptotic proteins (Bid, BAX, and caspase 9) and anti-apoptotic proteins (Bcl-2 and Bcl-XL) in skin tissues was detected by western blotting (Fig. 8B). As shown in Fig. 8D, the expression of *CTSF*, Bid, BAX, and caspase 9 was significantly lower in the ADSC group at both 4 and 12 weeks post-irradiation than in the control group at the same time points. In contrast, the expression of Bcl-2 and Bcl-XL was significantly higher in the ADSC group at both 4 and 12 weeks post-irradiation than in the control group at the same time points.

**Fig. 9:**
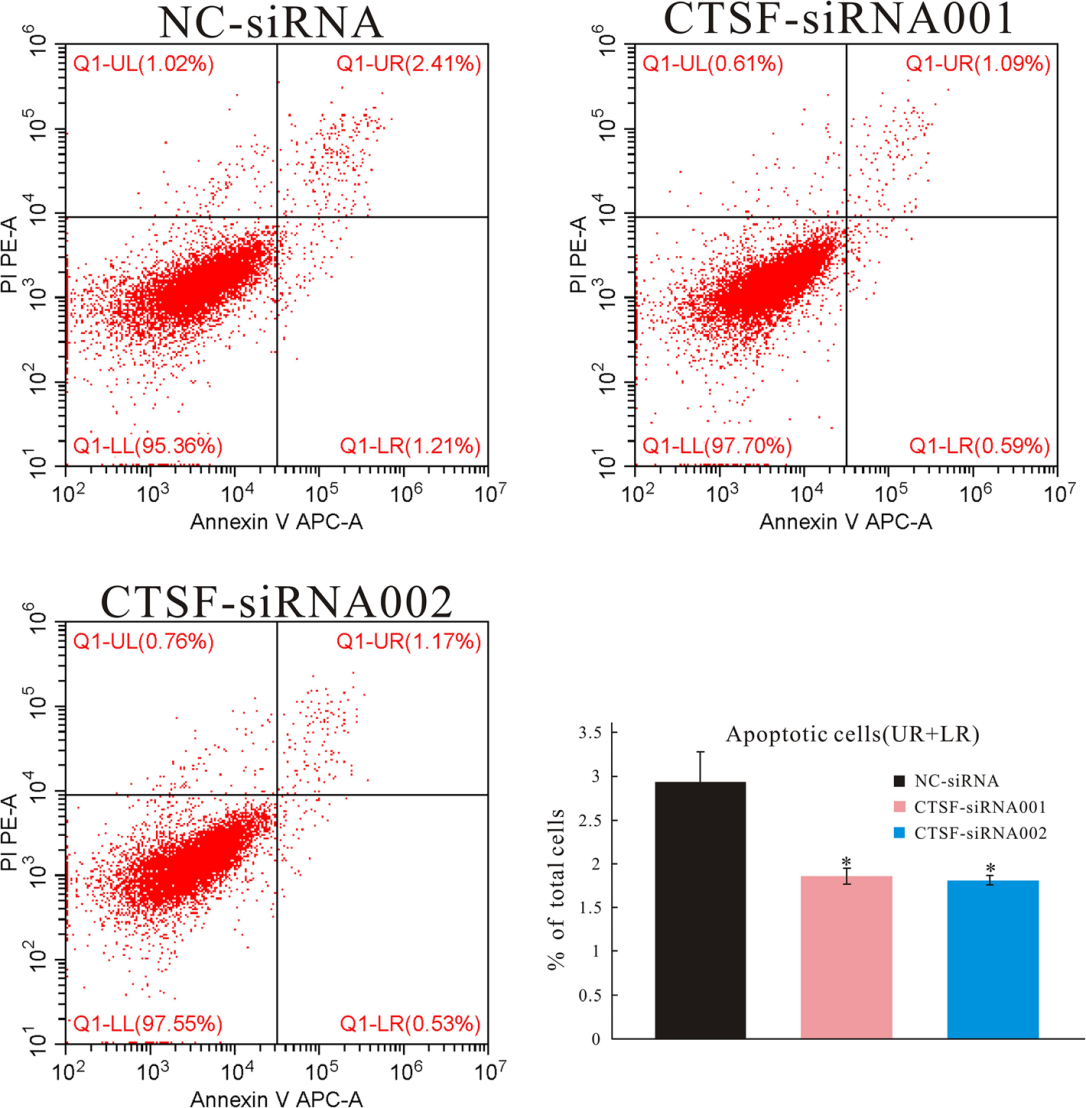
siRNAs were used to knock down *CTSF.* HaCaT cells were treated with NC-siRNA, *CTSF*-siRNA001, and *CTSF*-siRNA002 and were then harvested for the detection of apoptosis using flow cytometry. Upper left quadrant—necrotic cells; upper right quadrant—late apoptotic cells; lower left quadrant—viable cells; lower right quadrant—early apoptotic cells. The results are expressed as the mean ± SEM. *p < 0.05 vs. NC-siRNA group

**Fig. 10:**
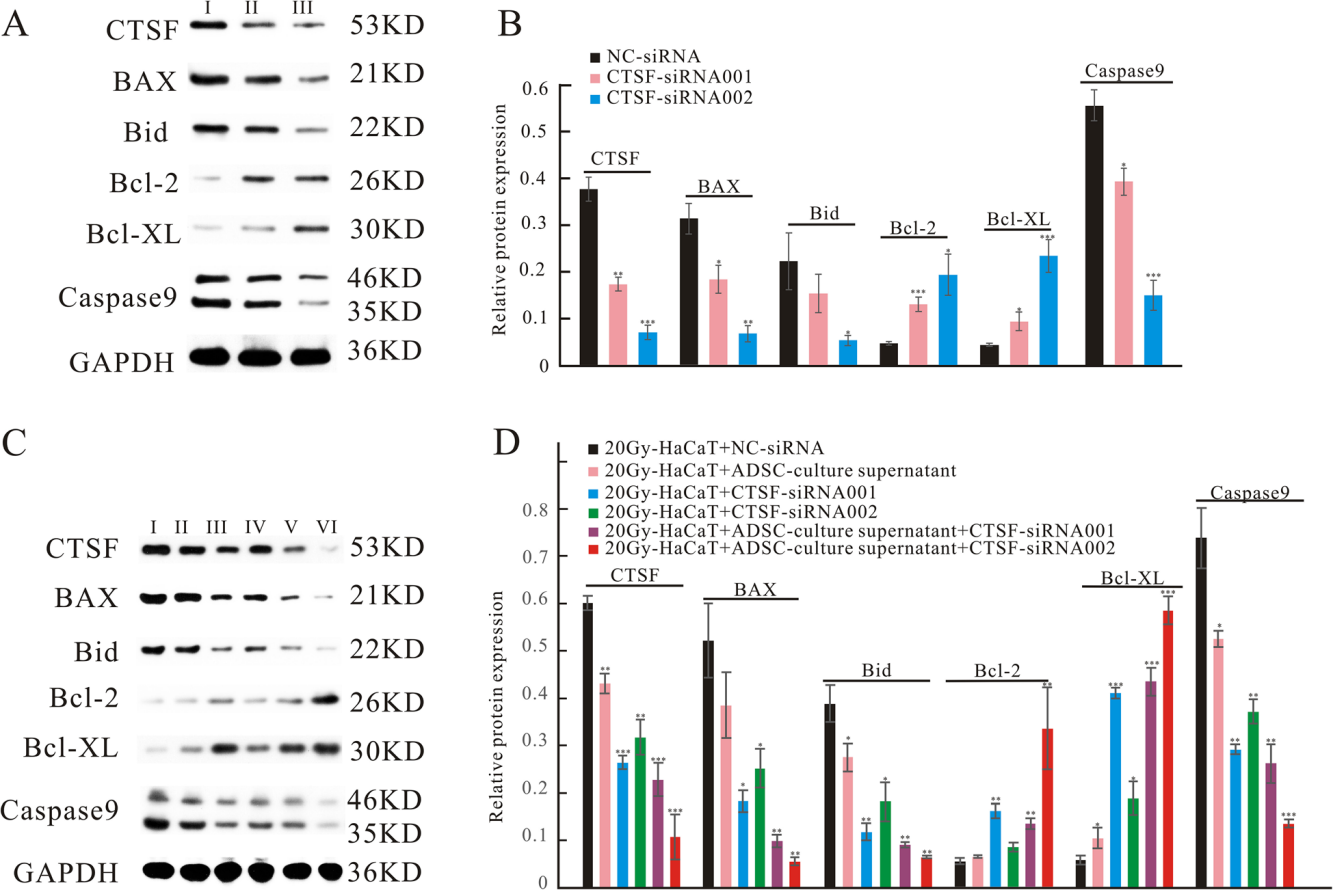
ADSC culture supernatant downregulates the expression of *CTSF*, BAX, Bid, and caspase 9 and upregulates that of Bcl-2 and Bcl-XL in a radiation-induced apoptosis cell model in vitro. Representative blots of *CTSF*, BAX, Bid, Bcl-2, Bcl-XL, and caspase 9 levels (**A**, **C**). GAPDH was used as a loading control. Quantitative analysis of protein levels (**B**, **D**). I–III in **A**: I, NC-siRNA; II, *CTSF*-siRNA001; III, *CTSF*-siRNA002. I–VI in **C**: I, 20Gy-HaCaT+NC-siRNA; II, 20Gy-HaCaT+ ADSC-culture supernatant; III, 20Gy-HaCaT+*CTSF*-siRNA001; IV, 20Gy-HaCaT+ *CTSF*-siRNA002; V, 20Gy-HaCaT+ADSC-culture supernatant+*CTSF*-siRNA001; VI, 20Gy-HaCaT+ADSC-culture supernatant+*CTSF*-siRNA002. The results are expressed as the mean ± SEM. *p < 0.05, **p < 0.01, ***p < 0.001 vs. NC-siRNA group

To demonstrate that ADSCs specifically target *CTSF*, siRNAs were used to knock down *CTSF*, radiation-induced apoptotic HaCaT cells were treated with ADSC culture supernatant, the apoptosis was analyzed by flow cytometry (Fig. 9), and the expression of *CTSF* and downstream proteins was evaluated by western blotting (Fig. 10A,C). As shown in Fig.9, the proportions of HaCaT cells in total apoptosis were lower in the *CTSF*-siRNA001 and *CTSF*-siRNA002 groups than in the NC-siRNA group. As shown in Fig. 10B, the expression of *CTSF*, BAX, Bid, and caspase 9 was downregulated in the *CTSF*-siRNA001 and *CTSF*-siRNA002 groups compared with that in the NC-siRNA group, whereas the expression of Bcl-2 and Bcl-XL was upregulated in the *CTSF*-siRNA001 and *CTSF*-siRNA002 groups when compared with that in the NC-siRNA group. As shown in Fig.10D, in the radiation-induced apoptosis cell model, the expression of *CTSF*, BAX, Bid, and caspase 9 was downregulated in cells treated with ADSC culture supernatant, *CTSF*-siRNA001, *CTSF*-siRNA002, or ADSC culture supernatant plus *CTSF*-siRNA001 or *CTSF*-siRNA002 when compared with that in the NC-siRNA group. The expression of Bcl-2 and Bcl-XL was upregulated in cells treated with ADSC culture supernatant, *CTSF*-siRNA001, *CTSF*-siRNA002, or ADSC culture supernatant plus *CTSF*-siRNA001 or *CTSF*-siRNA002 compared with that in the NC-siRNA group. These results suggested that ADSCs exhibit an anti-apoptotic effect by inhibiting *CTSF* expression.

## Discussion

In this study, we found that ADSCs decreased skin tissue damage, promoted hair follicle and sebaceous gland regeneration, and inhibited lymphocyte infiltration, apoptosis, and fibrosis formation, demonstrating that ADSCs alleviate acute and chronic radiation-induced dermatitis. Furthermore, ADSCs downregulated cathepsin F expression in a rat model of radiation-induced dermatitis.

ADSCs are one of the most promising stem cells as they are relatively easy to harvest and have a high proliferative ability. As ADSCs are not a homogeneous population, there is no unique single surface marker that can be used to characterize them. ADSCs can differentiate into various cell types; adipogenic, osteogenic, and chondrogenic three-line differentiation is the gold standard for characterization. In this study, ADSCs were isolated from inguinal adipose tissues and showed good adipogenic, osteogenic, and chondrogenic differentiation. Rigotti et al. reported that the stromal vascular fraction isolated from human lip aspirates alleviated chronic radiation-induced fibrosis clinically in 2005 [24]. Later, Sultan et al. found that fat grafting attenuated acute and chronic radiodermatitis in a murine model [25]. Human mesenchymal stem cells favor healing in the early phase of cutaneous radiation syndrome [26]. Huang et al. found that ADSCs accelerate radiation ulcer healing by increasing angiogenesis [27]. Human umbilical cord mesenchymal stem cells have been shown to improve irradiation-induced skin ulcer healing through keratin generation and epithelial cell proliferation [28]. Human fetal ADSC secretomes accelerated wound healing rate in rats with radiation-induced skin injury by promoting angiogenesis [29]. ADSCs have been shown to have antioxidant activity and protect cells from oxidative injury [30], and CD74^+^ ADSCs have been reported to possess anti-fibrotic activity [31]. Fibrosis is the main feature of chronic radiation-induced dermatitis, and our results showed that ADSCs reduce acute skin injury and fibrosis, which is consistent with previous studies. The data suggest that ADSCs can alleviate acute and chronic radiation-induced dermatitis; however, notably, their effects need to be further enhanced, and we aim to modify ADSCs to improve their effects in future.

Numerous clinical trials using ADSCs have been registered, including studies on their use in the treatment of diabetes, liver disease, fistulas, cardiovascular disease, limb ischemia, graft versus host disease, Crohn’s disease, and skin and bone defects. It is necessary to reveal the mechanisms underlying the effects of ADSCs in these pathologies. The ability of ADSCs to repair damaged tissue is mainly attributed to the autocrine and paracrine effects of their secretome, which comprises cytokines, extracellular proteins, and RNAs, including anti-inflammatory, immunomodulatory, antioxidant, and regeneration-stimulatory effects. It has been reported that ADSCs stimulate fibroblast and keratinocyte proliferation [32], and exosomes from human ADSCs promote skin fibroblast proliferation and migration [33]. ADSCs promote human umbilical vein endothelial cell proliferation, migration, and invasion by releasing miR-210 [34]. ADSC-conditioned medium promoted hair growth in vitro, ex vivo, and in vivo [35]. ADSCs can alleviate atopic dermatitis by regulating B cell maturation [36]. Chen et al. found that ADSCs potentially interfere with the formation of silicosis through anti-inflammatory and anti-apoptotic effects [20]. Our previous study showed that inflammation, apoptosis, hair follicle loss, and sebaceous gland loss are the main features of radiation-induced dermatitis in the acute stage [6], and the loss of follicles and sebaceous glands may be due to cell apoptosis. In 2004, Rehman et al. proposed that ADSCs have anti-apoptotic potential, which they exert through the secretion of anti-apoptotic factors [37]. To date, the constituents of ADSC-conditioned medium, which is a complex mixture, have not been fully identified. ADSC-secreted anti-apoptotic factors include insulin-like growth factor 1, superoxide dismutase 3, and miRNA-301a [38]. The exact factors from ADSCs that play roles in alleviating various diseases remain to be clarified. In this study, ADSCs inhibited lymphocyte infiltration and fibrosis formation, promoted hair follicle migration and regeneration, promoted sebaceous gland regeneration, and inhibited apoptosis in vivo and in vitro. In future, we aim to enhance the paracrine ability of ADSCs for anti-fibrosis and pro-regeneration through modification to improve their efficacy in the treatment of radiation-induced dermatitis. In addition, ADSCs not only downregulated *CTSF* in vivo and in vitro, but also regulated the expression of *CTSF*-downstream apoptosis-related proteins, and the change trends of these proteins were consistent with those after *CTSF* knockdown. These data indicated that ADSCs inhibit apoptosis through downregulating *CTSF* expression. However, which growth factors or microRNAs secreted from ADSCs directly target *CTSF* remain to be investigated.

## Conclusion

In summary, we examined the effects of ADSCs on acute and chronic radiation-induced dermatitis in a rat model. We found that ADSCs promote hair follicle and sebaceous gland regeneration and have anti-inflammatory, anti-fibrotic, and anti-apoptotic effects. Further, we found that ADSCs downregulate *CTSF* and downstream pro-apoptotic proteins (Bid, BAX, and caspase 9) and upregulate downstream anti-apoptotic protein (Bcl-2 and Bcl-XL) expression in vivo and in vitro. ADSCs protect against radiation-induced dermatitis, exhibit an anti-apoptotic effect by inhibiting *CTSF* expression, and may be a good therapeutic candidate to prevent the development of radiation-induced dermatitis.

## Data Availability

The datasets used and/or analyzed during the current study are available from the corresponding author on reasonable request.

## Abbreviations

ADSCs: Adipose-derived stem cells
IL: Interleukin
PBS: Phosphate-buffered saline
DMEM: Dulbecco’s modified Eagle’s medium
*CTSF*: Cathepsin F
